# A real-time measurement of general practice workload in the Republic of Ireland: a prospective study

**DOI:** 10.3399/bjgp20X710429

**Published:** 2020-06-02

**Authors:** Brendan Crosbie, Michael Edmund O’Callaghan, Stuart O’Flanagan, David Brennan, Gavin Keane, William Behan

**Affiliations:** Castle Street Surgery, Roscommon.; Department of General Practice, University of Limerick, Limerick.; Aranleigh Health Centre, Rathfarnham, Dublin.; Ballyhale Health Centre, Ballyhale, Kilkenny.; Morehampton Clinic, Dublin.; Trinity College Dublin GP Training Scheme, Dublin.

**Keywords:** computerised medical records systems, general practice, referral and consultation, planning techniques, real-time recording, workload

## Abstract

**Background:**

Demand for GP services in the Republic of Ireland (RoI) is increasing, and the resultant escalation in workload demands is an issue of growing concern. Accordingly, the accurate measurement and description of GP workload is essential to inform future healthcare planning.

**Aim:**

To provide a real-time measurement of GP workload with respect to hours worked and of proportional time expenditure on typical workload activities.

**Design and setting:**

A prospective study among GPs in the RoI that took place from January 2019 to March 2019.

**Method:**

Participants were invited to enrol in the study by direct email invitation and via notifications posted within GP-specific monthly journals; online forums; and a social media platform. Participants used a time-management software program to self-record workload activity in real time over 6 weeks.

**Results:**

In total, 123 GPs were included for final analyses with a total of 8930 hours of activity recorded. The mean duration of a two-session day (excluding break-time) was 9.9 hours (95% confidence interval [CI] = 9.7 to 10.0; interquartile range [IQR] 7.9 to 13.9). Of this time, 64% was spent on clinical consultations. In total, 25.4% of activity was recorded outside the hours of 9.00 am and 5.00 pm. An average of 12.4 face-to-face consultations were completed per session of activity. The mean duration of a 10-session week was greatest for the partner (50.8 hours; 95% CI = 49.8 to 51.9) and >55-year-old (50.8 hours; 95% CI = 49.3 to 52.2) demographics, relative to their respective colleagues.

**Conclusion:**

To the authors’ knowledge, this is the first study to provide an objective, accurate, and granular real-time measurement of GP workload in the RoI, demonstrating the significant volume and variety of work undertaken by GPs in the RoI.

## INTRODUCTION

GPs play a central role in the delivery of health care in the Republic of Ireland (RoI) completing approximately 19 million consultations per year.^1^ The demand for GP services is increasing both in the RoI and internationally as populations age and grow.^2^^,^^3^ The ability to meet future demand is an issue of increasing concern.^4^^–^^6^

Additional recruitment and improved retention of GPs are essential tenets for future healthcare planning,^7^^,^^8^ both of which may be compromised by concerns regarding GP workload. An increasing workload burden is the most common reason to consider emigration cited by newly qualified doctors and GP graduates in the RoI.^9^^,^^10^ It is also the most commonly referenced cause of burnout within the profession,^11^^–^^19^ which in turn can adversely affect patient care.^16^^,^^20^^–^^23^ Despite the growing recognition that GP workload must be maintained at manageable levels, knowledge of actual workload composition remains poor.

One of the earliest attempts to understand GP workload was undertaken in 1962 by means of a time-motion study.^24^ Attempts to measure this workload in subsequent decades have adopted retrospective survey-based approaches that have a number of potential limitations, including the possibility of introducing recall bias.^25^^,^^26^ Alternative approaches have involved direct observational measurements, which can be limited by scale given the need for on-site independent observers.^27^

Accordingly, there remains a distinct absence of large-scale accurate measurements of GP workload, not only for GPs in the RoI, but also internationally.

This study attempts to provide a comprehensive, objective, and accurate real-time measurement of GP workload from a large representative sample of GPs practising in the RoI.

## METHOD

This was a prospective study of daily GP workload recorded in real time among a population of GPs working in the RoI.

### Participant recruitment

Prospective participants were invited to enroll in the study by a variety of means, including: an email invitation circulated through GP-specific email groups, an article submitted to a GP-specific monthly publication (*Forum*), on GP-specific online forums (GPBuddy [https://www.gpbuddy.ie] and GP Forum [https://www.nationalgpforum.com]), and through the social media platform, Twitter.

### Data collection

A smartphone-based time-management software program (Time Doctor, version 2.5.01b157) was used by participants to self-record data in real time that automatically synchronised with a central project database. All recorded data were anonymised at the point of collection.

**Table:** How this fits in

GP workload is an issue of increasing concern. This study attempted to accurately measure this workload among GPs in the Republic of Ireland (RoI). The use of a real-time recording method aims to address the limitations of survey-based approaches for measuring workload activity. Data were recorded over a 6-week period with the results providing a comprehensive quantification of daily GP workload activity that, to the authors’ knowledge, has not been previously achieved elsewhere. The results have the potential to inform future planning and delivery of general practice in the RoI. This study also demonstrates an efficient and accurate method of capturing workload activity that could be employed in future measurements of workload.

Participants were requested to record a minimum of 10 sessions of working activity and were encouraged to record consecutive sessions of activity where possible. In accordance with the Irish College of General Practitioners’ definition, the study defined a session of working activity as any period of ≥3 hours spent undertaking GP-related activity, such that a full day of activity would consist of two sessions. Data were recorded over a 6-week period beginning in January 2019.

Work activity was recorded within the following pre-designated work-type categories:

- clinical consultation;
- clinical paperwork;
- telephone call;
- prescription;
- house call;
- break-time;
- Continuous Medical Education (CME)/research; and
- administrative work.

Out-of-hours activity was not included. Participants were required to submit demographic details pertaining to GP role, GP practice setting, age, and sex.

Participants were provided with a user guide to assist with software download and to use in addition to study definitions outlining each work category (see Supplementary Box S1 for details). An editing function was provided to amend erroneous recordings, and a support service was provided throughout the duration of data collection.

### Inclusion and exclusion criteria

Participants were required to be practising as a GP or GP registrar and registered with the Irish Medical Council for the duration of data recording; needed to possess a software-compatible smartphone device; and record ≥10 sessions or >39 hours of activity. Participants were excluded on the bases of not providing demographic details, recording an insufficient volume of data, and taking excessive time editing (>25% of total time).

### Statistical analysis

IBM SPSS Software (version 25.0) was used to analyse results using descriptive statistics. Break-time activity were excluded from analysis. Each participant’s data was standardised for 10 sessions before further analysis by dividing the total recorded activity by the total number of recorded sessions, and extrapolating for 10 sessions. Group comparisons of continuous variables were assessed using independent samples *t*-tests or Mann–Whitney *U*-tests as appropriate. Comparisons between demographic start and finish times were made using χ^2^ test or Fisher’s exact test, as appropriate. All tests were two-tailed, and a *P*-value of <0.05 was used to determine statistical significance.

### Participant feedback

On completion of data collection a SurveyMonkey questionnaire (see Supplementary Box S2 for details) was sent to all study participants, including those excluded from the final data analysis, to measure the perceived ease of software download and use. Participants were also invited to submit a retrospective count of the number of consultations completed during the dates of data recording.

## RESULTS

### Participant sample and demographics

**Figure 1.**
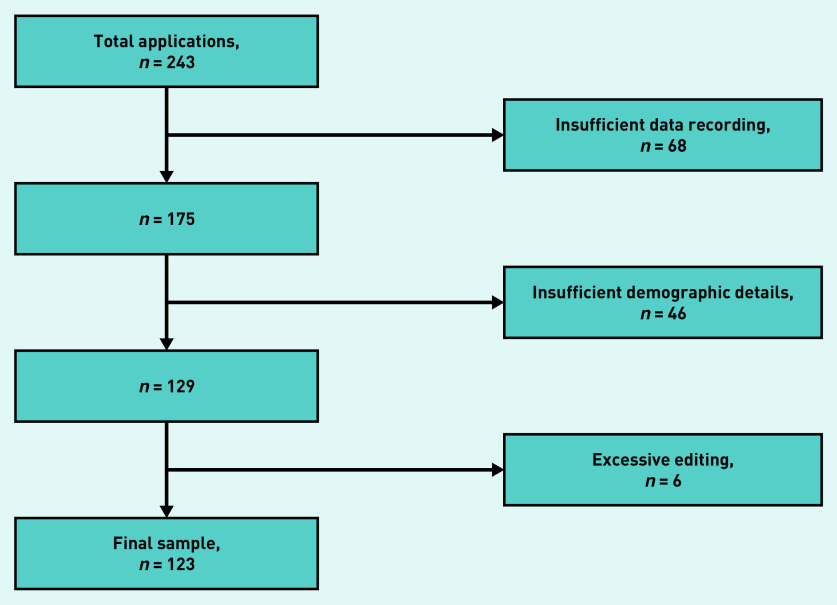
***Flow chart demonstrating sample size following the application of exclusion criteria.***

A total of 243 participants were enrolled in the study, of which 123 (50.6%) were included for final data analysis following the application of exclusion criteria (Figure 1). Sample demographics are demonstrated in Table 1.

**Table 1.** Characteristics of the sample population, *N* = 123

**Characteristic**	**Partner (%)**	**Assistant (%)**	**Registrar (%)**	**Urban (%)**	**Mixed (%)**	**Rural (%)**
**Sex**						
Male	44 (52.4)	9 (50.0)	7 (33.3)	34 (47.2)	11 (44.0)	15 (57.7)
Female	40 (47.6)	9 (50.0)	14 (66.7)	38 (52.8)	14 (56.0)	11 (42.3)

**Age, years**						
<45	16 (19.0)	15 (83.3)	21 (100)	29 (40.3)	9 (36.0)	14 (53.8)
45–54	35 (41.7)	3 (16.7)	0 (0)	23 (31.9)	8 (32.0)	7 (26.9)
≥55	33 (39.3)	0 (0)	0 (0)	20 (27.8)	8 (32.0)	5 (19.2)

**Location**						
Urban	47 (56.0)	12 (66.7)	13 (61.9)	72 (58.5)	—	—
Mixed	21 (25.0)	2 (11.1)	2 (9.5)	—	25 (20.3)	—
Rural	16 (19.0)	4 (22.2)	6 (28.6)	—	—	26 (21.1)

**Role**						
Partner	84 (68.3)	—	—	—	—	—
Assistant	—	18 (14.6)	—	—	—	—
Registrar	—	—	21 (17.1)	—	—	—

### Total data recorded

A total of 8930 hours recorded over 1698 sessions of activity (mean 13.8 sessions per user; interquartile range [IQR] 10.0 to 16.0) and 1158 days (1109 weekdays and 49 weekend days) were included for analysis.

### Duration of sessions and proportional workload

**Figure 2.**
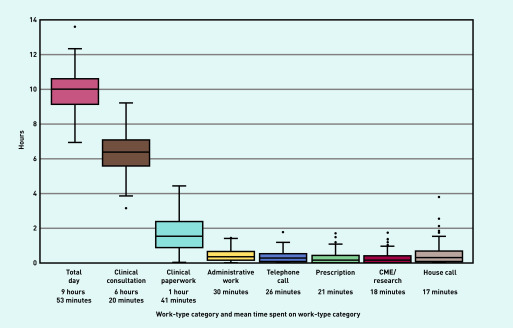
***Boxplot demonstrating the breakdown of daily activity according to work-type categories for the total population. CME = Continuous Medical Education.***

The mean duration of 10 sessions of activity excluding break-time was 49.4 hours (95% confidence interval [CI] = 48.4 to 50.4; range 39.4 to 69.5), which varied by demographic group (Table 2). The mean duration of a two-session day following the exclusion of break-time was 9.9 hours (95% CI = 9.7 to 10.0; range 7.9 to 13.9) with the breakdown of daily activity by work-type demonstrated in Figure 2.

**Table 2.** Time spent completing 10 sessions and the proportional time expenditure on different working activities

**Demographic**	***N***	**Total time, mean hours, %**	**Clinical consultation, hours (%)**	**Clinical paperwork, hours (%)**	**Telephone call, hours (%)**	**Prescription, hours (%)**	**House call, hours (%)**	**CME/research, hours (%)**	**Administrative work, hours (%)**
**Total Sample**	123	49.4	31.64 (64.0)	8.36 (16.9)	2.20 (4.5)	1.73 (3.5)	1.43 (2.9)	1.52 (3.1)	2.52 (5.1)

**Role**									
Partner	84	50.8	31.77 (62.5)	8.50 (16.7)	2.39 (4.7)	1.71 (3.4)	1.63 (3.2)	1.61 (3.2)	3.21 (6.3)
Assistant	18	47.5	33.97 (71.5)	6.61 (13.9)	1.51 (3.2)	1.64 (3.5)	1.36 (2.9)	0.95 (2.0)	1.47 (3.1)
Registrar	21	45.4	29.13 (64.2)	9.31 (20.5)	2.02 (4.4)	1.88 (4.1)	0.71 (1.6)	1.67 (3.7)	0.68 (1.5)

**Location**									
Urban	72	49.6	31.56 (63.6)	8.95 (18.0)	2.08 (4.2)	1.82 (3.7)	1.22 (2.5)	1.30 (2.6)	2.67 (5.4)
Mixed	25	49.8	33.28 (66.8)	7.12 (14.3)	1.97 (4.0)	1.57 (3.2)	1.61 (3.2)	1.90 (3.8)	2.35 (4.7)
Rural	26	48.5	30.29 (62.4)	7.93 (16.3)	2.73 (5.6)	1.63 (3.4)	1.87 (3.9)	1.77 (3.6)	2.29 (4.7)

**Age, years**									
<45	52	47.7	32.24 (67.6)	7.91 (16.6)	1.90 (4.0)	1.48 (3.1)	0.90 (1.9)	1.30 (2.7)	1.94 (4.1)
45–54	38	50.6	30.42 (60.1)	8.78 (17.3)	2.51 (5.0)	2.07 (4.1)	1.51 (3.0)	1.95 (3.9)	3.39 (6.7)
≥55	33	50.8	32.11 (63.3)	8.60 (16.9)	2.31 (4.6)	1.73 (3.4)	2.18 (4.3)	1.38 (2.7)	2.45 (4.8)

**Sex**									
Male	60	49.4	31.60 (64.0)	7.53 (15.2)	2.17 (4.4)	1.65 (3.3)	2.05 (4.1)	1.66 (3.4)	2.75 (5.6)
Female	63	49.4	31.69 (64.1)	9.16 (18.5)	2.22 (4.5)	1.81 (3.7)	0.84 (1.7)	1.39 (2.8)	2.30 (4.7)

CME = Continuous Medical Education.

Partners worked more hours over 10 sessions (50.8 hours; 95% CI = 49.8 to 51.9) when compared with both assistants (47.5 hours; 95% CI = 44.8 to 50.3; *P* = 0.025) and registrars (45.4 hours; 95% CI = 42.9 to 48.0; P = 0.001) (Table 2). Similarly, both those aged 45–54 years (50.6 hours; CI = 49.0 to 52.2; P = 0.005) and ≥55 years (50.8 hours; CI = 49.3 to 52.2; *P* = 0.004) worked a greater number of hours compared to those aged <45 years (47.7 hours; CI = 45.9 to 49.4) (Table 2).

Clinical consultations represented the greatest workload undertaking for all demographic groups (Table 2). Assistants spent more time completing clinical consultations (34.0 hours; CI = 30.9 to 37.0; P = 0.045) relative to their non-assistant counterparts (31.2 hours; CI = 30.3 to 32.2; P = 0.045), while partners spent more time on administrative activity (3.2 hours; CI = 2.5 to 3.9; P = 0.001) and telephone calls (2.4 hours; CI = 2.0 to 2.8; P = 0.035) than the aggregated non-partner demographic time expenditure on either activity (1.1 hours; CI = 0.4 to 1.7 versus 1.8 hours; CI = 1.2 to 2.4).

Those aged ≥55 years spent a greater proportion of their time on house calls (2.2 hours; CI = 1.4 to 3.0; P = 0.009) relative to their younger counterparts (1.2 hours; CI = 0.8 to 1.5, P = 0.009) (Table 2). Similarly, both rural (1.9 hours; CI = 1.1 to 2.6; P = 0.049) and male (2.1 hours; CI = 1.5 to 2.6; P = 0.001) GPs spent a greater proportion of time on house calls than their urban (1.2 hours; CI = 0.8 to 1.6) and female (0.8 hours; CI = 0.5 to 1.2) counterparts, respectively.

### Distribution of hours worked

Of the total number of hours recorded, 74.6% (6659 hours) were on weekdays between 9 am and 5 pm. Of the remaining 2271 hours, 17.5% were recorded before 9 am, 77.8% after 5 pm, and 4.7% at weekends. Partners completed 91.4% of the total hours recorded during weekends, with 84.3% of this time spent on non-consultation activities.

Recording started before 9 am and finished after 5 pm on 52.6% and 72.7%, respectively, of the total number of weekdays recorded, with the respective proportions varying by demographic group (Table 3).

**Table 3.** Recording start and finish times of the total number of days recorded

**Demographic**	***N***	**Recorded start time, %**			**Recorded finishing time, %**						
	
		**<9.00 am**	**<8.00 am**	**<7.00 am**	**>5.00 pm**	**>6.00 pm**	**>7.00 pm**	**>8.00 pm**	**>9.00 pm**	**>10.00 pm**	**>11.00 pm**
**Total Sample**	1109	52.6	10.5	1.9	72.7	33.8	18.7	7.9	7.0	5.1	3.8

**Role**											
Partner	815	57.5	11.7	2.5	73.6	60.9	35.9	20.8	13.9	7.9	3.5
Assistant	134	33.3	4.8	0.0	66.3	40.3	16.9	8.2	6.4	3.9	1.2
Registrar	160	43.1	9.0	0.0	69.3	37.1	14.3	6.5	4.0	0.4	0.0

**Location**											
Urban	642	49.7	11.3	1.5	71.8	54.2	30.7	17.6	10.8	5.6	2.2
Mixed	209	52.1	7.4	2.6	73.5	54.1	28.3	11.8	8.6	3.8	2.4
Rural	258	60.6	10.8	2.5	71.8	57.6	32.5	20.2	15.1	10.0	4.0

**Age, years**											
<45	345	48.3	9.1	0.9	71.1	46.0	20.2	10.9	7.6	4.5	2.6
45–54	342	56.5	15.2	3.6	73.4	60.0	39.9	22.3	15.2	8.3	3.1
≥55	422	53.7	7.4	1.4	71.9	60.3	32.6	18.6	12.0	6.5	2.5

**Sex**											
Male	570	52.6	8.3	2.5	74.5	59.7	33.0	19.0	12.4	7.4	3.4
Female	539	52.7	12.8	1.3	69.7	49.8	27.8	14.8	10.2	5.2	1.9

### Participant feedback

A total of 66 participants (53.7%) completed the feedback questionnaire, of whom 45 (68.2%) were GP partners, 12 (18.2%) were assistant GPs, and nine (13.6%) were GP registrars. Responder ages mirrored those of the full sample population, with 28 (42.4%) being <45 years old, 19 (28.8%) being between 45–54 years old, and 19 (28.8%) being ≥55 years old. In total, 53 (80.3%) responders recorded ≥10 sessions of activity with the remaining 13 (19.7%) recording <10 sessions.

Eighty-nine per cent of responders reported that the use of the software was either ‘very easy’ or ‘easy’, with the remaining 11% reporting ‘average’ ease of use. None of the responders rated the use of the software as ‘difficult’ or ‘very difficult’.

### Consultation numbers

Twenty-six GPs provided data for the number of completed consultations, of whom 14 (53.8%) were partners, 6 (23.1%) were assistants, and 6 (23.1%) were registrars; 17 participants (65.4%) were female. A total of 3906 consultations were recorded over 316 sessions with an average of 12.4 consultations being completed per session of activity. This figure varied among demographic groups with the assistant demographic completing more consultations on average per session (13.3) compared with the partner (12.8) and registrar (11.2) demographics. The average duration of a consultation was 14 minutes 53 seconds, with the partner demographic (14 minutes 36 seconds) recording the shortest average consultation length as compared with the assistant (15 minutes 42 seconds) and registrar (14 minutes 59 seconds) demographics.

## DISCUSSION

### Summary

This study highlights the significant number of hours being worked by GPs across all demographics in the RoI, in particular by older GPs and those in more senior roles. The pattern of longer session lengths and later finishing times observed among the ≥ 55-year-old age category highlights the challenge of replacing this cohort of GPs approaching retirement age. This is of even greater concern given that approximately one-third of GPs in the RoI are ≥55 years old.^25^^–^^27^

These findings amplify concerns for future retention and recruitment efforts, given the need for new GP graduates to meet both the current workload undertaken by their senior counterparts and future increases of this workload. It should also be noted that this study does not include the increasing out-of-hours workload burden.^2^

The variable availability of out-of-hour services in rural locations might explain some of the differences observed for total hours worked, later finishing times, and time spent on house calls between different GP demographics. This raises concerns for the future sustainability of rural general practice in its current form.

The results also demonstrate the significant proportion of workload that does not involve face-to-face on-site consultations. Clinical paperwork is the largest contributor to this non-consultation workload. It is likely that some proportion of these tasks could be transferred to non-doctor professionals within the general practice team; however, the variable availability and affordability of additional practice staff may limit the feasibility of such transfers.

Irish general practice is partly funded by a fee-per-service remuneration system; however, one-third of total time recorded in the current study was spent on non-consultation activities. This highlights the significant unseen and often unappreciated workload burden that likely exists in every GP system, regardless of funding mechanism.

Despite the significant non-consultation workload burden, the subsample results demonstrate that GPs complete >25 face-to-face consultations on average per two-session day. The observed average consultation length of 14 minutes 53 seconds is in keeping with the 15-minute duration of clinical consultation recommended by the Royal College of General Practitioners,^28^ but is longer than the average consultation lengths observed in previous studies.^4^^,^^29^ These differences may be explained by variations in recording methods, definitions of consultations, and consulting styles and cultures. The longer consultation time is to be welcomed and should be protected by future workload-planning strategies to optimise patient safety, particularly in more complex consultations.^30^ However, it is important to note that the current results are from a small subset analysis and do not allow for intra-sample differences in consultation styles.

### Strengths and limitations

Analysis of independently recorded GP daily activity produced a detailed picture of current GP workload in the RoI. The large volume of hours recorded and relatively tight CIs allay concerns regarding reporting bias.

The present study sample can be considered representative of the national population, as the demographics of the participant population correspond to those observed in previous studies of general practice in the RoI.^25^^,^^27^ The final 123 participants represent nearly 3% of the approximate 4350 GPs practising in the RoI.

The results of the participant feedback survey, from a subsample population representative of the full sample group, suggest that the use of time-management software is a user-friendly and time-efficient method of large-scale measurement of GP activity and workload.

The present study has a number of potential limitations, including that participants were not directly observed during data recording and the potential of introducing selection bias during sample recruitment in favour of participants with a greater technological acumen. Furthermore, break-time activity was excluded from all analyses as a subset of participants stopped recording all activity during breaks rather than record break-time as a specific activity. This would have the potential effect of underestimating the overall number of hours worked. Telephone activity was not subdivided into specific call types, and hence telephone consultations are not included in the total number of consultations recorded.

Participation bias is a potential limitation of the study given that some prospective participants may not have enrolled because of concerns about any additional workload burden arising from participation.

Fifty-seven per cent of the participants that were excluded for the final data analysis were removed as they failed to record a sufficient volume of data. Given that the feedback questionnaire was completed both by included and excluded participants, and that its results suggested that software download and use was user friendly, it is likely that existing time pressures limited some participants’ capacity to adopt an additional activity that was not directly linked to patient care.

An efficient and user-friendly method of recording consultation numbers in real time may have resulted in a larger subsample population than was produced by the retrospective email approach adopted.

The present study is specific to general practice in the RoI, which may limit generalisation to non-Irish populations.

The present study does not include a measure of practice nurse workload, which could provide further detail on practice workload burdens.

### Comparison with existing literature

There is a relative paucity of research examining GP workload, and still less that examines the topic in the detail set out in the present study. The total number of hours recorded is greater than that of previous studies.^25^^,^^26^ The 64% proportional time expenditure on clinical consultations is similar to previously reported results,^25^^,^^26^ although the present study demonstrated some variance among different demographic groupings. The present study also provides subcategorised results and demographic detail not published elsewhere, including start and finish times and data pertaining to the GP registrar demographic. To the authors’ knowledge, this is the first study of its type to undertake a real-time participant-recorded measurement of workload activity; as a result, the method of data collection is without direct comparison.

### Implications for research and practice

The results of the present study highlight the large volume and diversity of workload undertaken by Irish GPs, and underlines the future challenge of replacing older GPs. It also raises concerns regarding the future sustainability of general practice in rural settings. This study may inform future healthcare planning, in particular recruitment and retention strategies, by outlining current challenges arising from GP workload burden.

The volume and detail of data recorded using this methodology are encouraging, and, in conjunction with the results from the participant feedback survey, support the use of technology-based data collection methods for workload measurements.

This study accurately demonstrates the considerable workload undertaken in Irish general practice, including significant time being spent on non-consultation activity. In addition to the rising demands on GP services in the RoI, the current workload being shouldered by older GPs will need to be distributed among younger GPs and the wider general practice team in order to meet future patient care needs. The present study also demonstrates the applicability of modern technology to large-scale independent research in the field of general practice, which has traditionally been compromised by limitations of scale.
